# History of autoimmune disease is associated with impaired survival in multiple myeloma and monoclonal gammopathy of undetermined significance: a population-based study

**DOI:** 10.1007/s00277-016-2859-8

**Published:** 2016-11-02

**Authors:** Ebba K. Lindqvist, Ola Landgren, Sigrún H. Lund, Ingemar Turesson, Malin Hultcrantz, Lynn Goldin, Magnus Björkholm, Sigurdur Y. Kristinsson

**Affiliations:** 1 Department of Medicine, Division of Hematology, Karolinska University Hospital and Karolinska Institutet, 171 76 Stockholm, Sweden; 2 Department of Medicine, Myeloma Service, Memorial Sloan-Kettering Cancer Center, New York, NY USA; 3 Faculty of Medicine, University of Iceland, Reykjavik, Iceland; 4 Department of Hematology and Coagulation Disorders, Skåne University Hospital, Malmö, Sweden; 5 Division of Cancer Epidemiology and Genetics, National Cancer Institute (NCI), National Institutes of Health (NIH), Bethesda, MD USA

**Keywords:** Autoimmunity, Multiple myeloma, MGUS, Survival, Population-based

## Abstract

Multiple myeloma (MM) is a plasma cell disorder preceded by monoclonal gammopathy of undetermined significance (MGUS). Incidence of MM and MGUS is higher among patients with autoimmune disease. The aim of this study was to determine whether a history of autoimmunity has an impact on survival in MM and MGUS. Using high-quality national Swedish registries, we identified 8367 patients with MM, 18,768 patients with MGUS, and 110,251 matched control subjects, and obtained information on previous autoimmune disease in patients and controls. Cox regression was used to calculate hazard ratios (HRs) for overall survival with 95 % confidence intervals (CIs). In patients with MM and a prior autoimmune disease, the risk of death was significantly increased, HR = 1.2 (95 % CI 1.2–1.3) compared to MM patients with no history of autoimmunity. In MGUS patients, a prior autoimmune disease was associated with a significantly 1.4-fold elevated risk of death (95 % CI 1.3–1.4). When analyzing different types of autoimmune diseases, a history of ulcerative colitis had a stronger impact on survival in MM than in controls. Our findings that a history of autoimmune disease has a negative impact on survival in MM and MGUS could be due to shared underlying common genetic factors, or that patients with a history of autoimmunity develop more severe cases of MM and MGUS, or cumulative comorbidity in the individual. Our results suggest that more attention should be paid to comorbidity as a prognostic factor in MGUS and MM, and underlines the need for studies aimed at tailoring therapy according to comorbidity.

## Introduction

Multiple myeloma (MM) is a chronic plasma cell disorder, characterized by a monoclonal proliferation of plasma cells in the bone marrow coupled with an overproduction of monoclonal (M-) protein [1]. Clinical manifestations of MM include osteolytic lesions, anemia, renal failure, and hypercalcemia [2]. Monoclonal gammopathy of undetermined significance (MGUS) is an asymptomatic, pre-malignant condition with an average risk of progression to MM or other lymphoproliferative disorders of 1 % per year [3].

The etiology of MM and MGUS is largely unknown. Familial risk factors have been identified, such as an increased risk of MM and MGUS in first-degree relatives of patients with these disorders [4–7]. This, together with ethnic disparities in the incidence patterns [8, 9], suggests a role for genetic factors in the etiology. Furthermore, high age and male gender have been found to be risk factors for MGUS, as have environmental risk factors such as exposure to pesticides and herbicides [10–12].

Autoimmune diseases include a variety of conditions, which jointly affect 5 to 10 % of the population [13]. Autoimmune diseases are characterized by increased activation of T or B cells toward own antigens (autoreactivity), causing local or systemic symptoms [14]. A history of autoimmunity increases the risk of certain malignancies [15–19], possibly due to chronic stimulation of the immune system; however, the complete underlying mechanisms are unknown. We and several investigators have shown that a personal history of autoimmune disease is associated with a significantly increased risk of MGUS and to some extent MM [20–22]. Our research group recently presented results showing a family history of autoimmune disease to be associated with a significantly increased risk of MGUS [20], suggesting a common genetic susceptibility between autoimmunity and plasma cell disorders.

Autoimmune disease is a predictor of poor survival in the general population, especially among women [23, 24]. A history of a few specific autoimmune diseases has been found to increase mortality in patients with certain digestive tract cancers [17], but does not seem to influence prognosis in other cancer types, e.g., lung cancer [18]. In a large study on patients with Hodgkin lymphoma, a prior diagnosis of autoimmune disease was associated with poorer survival [25]. In a smaller study on Swedish MM patients, the effect of 33 different autoimmune diseases on survival was analyzed, and only a history of rheumatic fever was associated with a decreased survival, although results were limited due to small numbers [22].

To increase knowledge in this field, we conducted a population-based study to determine whether a personal history of autoimmune disease has an impact on survival in MM and MGUS.

## Methods

### Registries, patients, and control subjects

Patients in Sweden with MM are treated by physicians at hospital-based hematology or oncology centers. All physicians in Sweden are obliged to report each case of incident cancer to the nationwide Swedish Cancer Register [26]. The completeness and diagnostic accuracy of the register is high (>93 %) for MM [27]. We identified all patients with a diagnosis of MM diagnosed from January 1, 2000, through December 31, 2013, in the nationwide Swedish Cancer Register. We established an MGUS cohort that has been described previously [20] consisting of MGUS patients retrieved through a national network, as well as through the Swedish Inpatient and Outpatient Registers, which have a high level of coverage and accuracy [28, 29]. MGUS patients diagnosed from January 1, 1988 through December 31, 2013 were included.

For all included patients, we obtained information on sex, date of birth, and date of diagnosis where the diagnosis was made. For MGUS patients, information on M-protein type and concentration was collected, where this was available. For each MM and MGUS patient, four population-based control subjects matched by sex, year of birth, and county of residence were chosen randomly from the Swedish Register of Total Population database. The control subjects had to be alive and free of any preceding hematologic malignancy at the time of MM or MGUS diagnosis of the corresponding case.

We obtained information on occurrence and date of autoimmune disease in patients and in controls from the Swedish Inpatient Register from 1964 and onwards. The conditions included in the analyses were equivalent to previously published studies (Appendix 1) [20, 30].

Information on survival was gathered from the Swedish Cause of Death Register. End of follow-up was December 31, 2013.

### Statistical analysis

xWe used the Kaplan-Meier method with log-rank test and regression models to compare outcome among patients and controls with and without autoimmune disease. Specifically, we calculated hazard ratios (HRs) and 95 % confidence intervals (CIs) with Cox proportional hazards models that were adjusted for age, year of diagnosis, and sex. In a sensitivity analysis, each MM or MGUS case was paired with a control, matching on age of diagnosis, and the risk for mortality was calculated using Cox proportional hazards model as stated above.

To avoid the possibility of autoimmune disease being discovered more often in cases than in controls due to the diagnostic work up of a plasma cell disorder, we excluded all autoimmune disease diagnosed less than 1 year prior to diagnosis of MM or MGUS. In order to investigate whether exposure time in addition to the presence of autoimmune disease had an impact on outcome, we included the duration of exposure in a separate model.

We performed analyses on seven specific autoimmune diseases previously found to increase the risk of MM and/or MGUS [20]; rheumatoid arthritis, pernicious anemia, chronic rheumatic heart disease, ulcerative colitis, polymyalgia rheumatica, giant cell arteritis, and psoriasis. Among MGUS patients, we also performed analyses on M-protein concentration (all isotypes combined) and by M-protein isotype where IgM MGUS was analyzed separately and IgG and IgA MGUS combined since IgM MGUS and non-IgM MGUS appear to be clinically distinct with regards to progression [31].

## Results

A total of 8367 patients with MM and 18,768 patients with MGUS that were diagnosed from January 1, 2000 and from January 1, 1988 through December 31, 2013, respectively, were included in the study, as well as 33,577 matched control subjects for MM and 76,674 matched control subjects for MGUS (Table 1). The median age at diagnosis was 72 years for both MM and MGUS patients.

**Table 1:** Patient characteristics

	MM^a^ patients	MM controls	MGUS^b^ patients	MGUS controls
No^c^. in total	8367	33,577	18,768	76,674
Males no. (%)	4636 (55)	18,606 (55)	9765 (52)	39,928 (52)
Females no. (%)	3731 (45)	14,971 (45)	9003 (48)	36,746 (48)
Median age at diagnosis, years (range)	71 (31–97)		73 (30–101)	
No. with AI^d^ (%)	1378 (16)	4380 (13)	4032 (21)	9046 (12)
Males (%)	688 (50)	2210 (51)	1990 (49)	4498 (50)
Females (%)	690 (50)	2170 (49)	2042 (51)	4548 (50)
Median age at diagnosis, years (range)	75 (32–98)		76 (31–101)	
Median age at AI diagnosis, years (range)	70 (16–97)	69 (7–97)	66 (10–96)	68 (10–99)
No. without AI (%)	6989 (84)	29,197 (87)	14,736 (79)	67,628 (88)
Males (%)	3948 (57)	16,396 (56)	7775 (53)	35,430 (52)
Females (%)	4202 (43)	12,801 (45)	8262 (47)	32,198 (47)
Median age at diagnosis, years (range)	70 (31–96)		71 (30–99)	

^a^ *MM* multiple myeloma

^b^ *MGUS* monoclonal gammopathy of undetermined significance

^c^ *No*. number

^d^ *AI* autoimmune disease

**Fig. 1:**
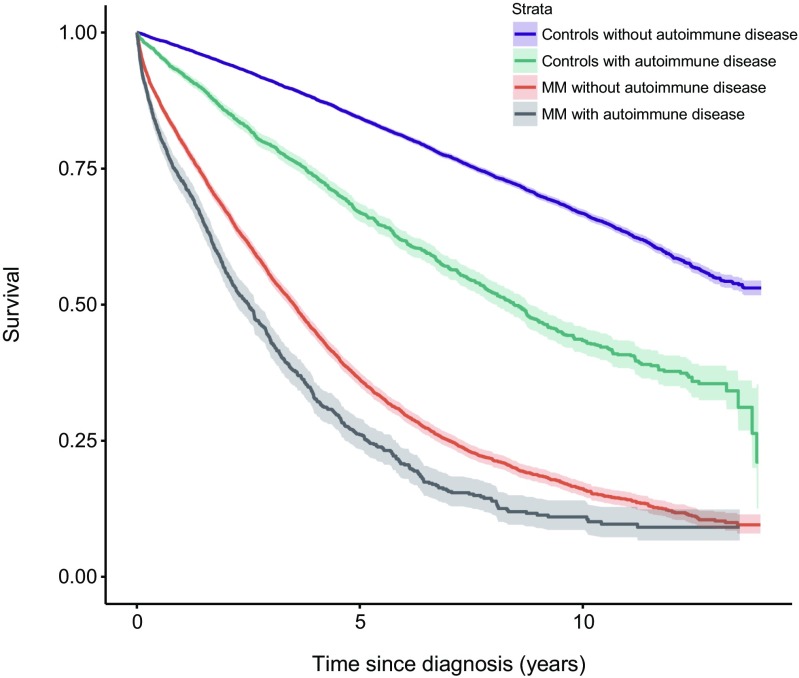
Survival in patients with multiple myeloma (*MM*), with and without a personal history of autoimmune disease, compared to controls with and without a personal history of autoimmune disease

A history of autoimmune disease was found in 1378 MM (16 %) patients and in 4380 MM controls (13 %). Compared to MM patients with no history of autoimmune disease, patients with MM and a prior history of autoimmunity had a significantly increased risk of death (HR = 1.2, 95 % CI 1.2–1.3). The increased risk was similar in males (HR = 1.3, 95 % CI 1.1–1.4) and in females (HR = 1.2, 95 % CI 1.1–1.3). Compared to controls without prior autoimmune disease, MM controls with a history of autoimmunity had a significantly increased risk of death (HR = 1.8, 95 % CI 1.7–1.9) (Fig. 1, Table 2). In a sensitivity analysis, we excluded all individuals with a previous diagnosis of cancer, and found essentially the same results (data not shown).

**Table 2:** History of AI and survival in MM and MGUS

	MM^a^		MGUS^b^	
	HR^c^	95 % CI^d^	HR	95 % CI
Females				
Groups: MM/MGUS + AI^e^ vs. MM/MGUS + no AI No. deaths/subjects in each group:	1.2 462/690 1947/3041	1.1–1.3	1.4 936/2042 3415/6961	1.3–1.5
Controls + AI vs. controls + no AI No. deaths/subjects in each group:	1.9 719/2170 2679 /12,801	1.7–2.0	1.7 1722/4548 11,239/32,198	1.6–1.8
Males				
MM/MGUS + AI vs. MM/MGUS + no AI No. deaths/subjects in each group:	1.3 448/688 2471/3948	1.1–1.4	1.4 950/1990 4199/7775	1.3–1.5
Controls + AI vs. controls + no AI No. deaths/subjects in each group:	1.8 811/2210 3903/16,396	1.6–1.9	1.6 1878/4498 13,798/35,430	1.6–1.7
Overall				
MM/MGUS + AI vs. MM/MGUS + no AI No. deaths/subjects in each group:	1.2 910/1378 4418/6989	1.2–1.3	1.4 1886 /4032 7614/14,736	1.3–1.4
Controls + AI vs. controls + no AI No. deaths/subjects in each group:	1.8 1530 /4380 6582/29,197	1.7–1.9	1.7 3600/9046 25,037/67,628	1.6–1.7

^a^ *MM* multiple myeloma

^b^ *MGUS* monoclonal gammopathy of undetermined significance

^c^ *HR* hazard ratio

^d^ *CI* confidence interval

^e^ *AI* autoimmune disease

**Fig. 2:**
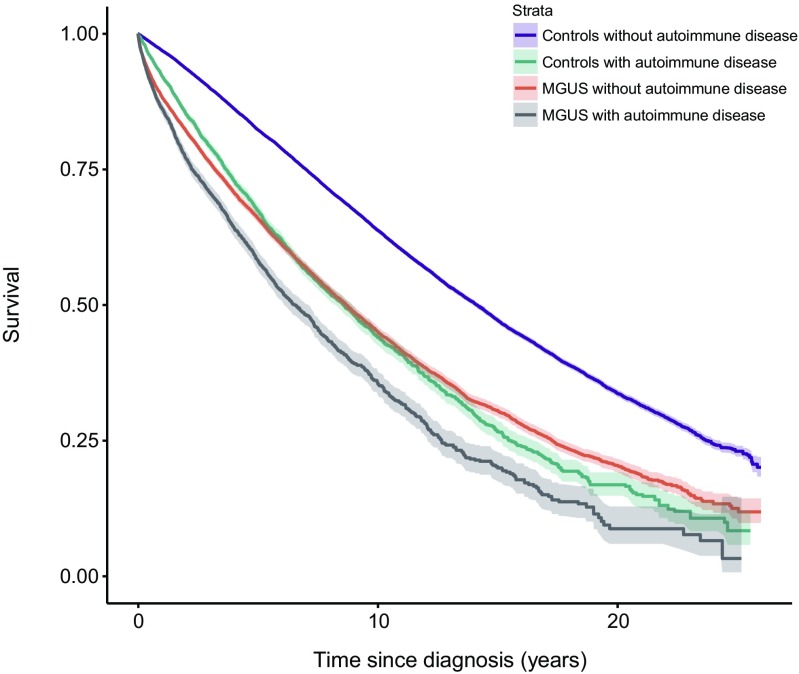
Survival in patients with monoclonal gammopathy of undetermined significance (*MGUS*), with and without a personal history of autoimmune disease, compared to controls with and without a personal history of autoimmune disease

A history of autoimmune disease was found in 4032 MGUS (21 %) patients and in 9046 MGUS controls (12 %). Compared to MGUS patients with no history of autoimmune disease, patients with MGUS and a prior history of autoimmunity had a significantly increased risk of death (HR = 1.4, 95 % CI 1.3–1.4). The results were the same for both females and males. Compared to controls without prior autoimmune disease, MGUS controls with prior autoimmunity had a significantly increased risk of death (HR = 1.7, 95 % CI 1.6–1.7) (Fig. 2, Table 2).

By a likelihood ratio test, the difference in effects of autoimmune disease in MM and MGUS patients compared to that in controls was statistically significant. The duration of exposure to autoimmune disease did not have a significant effect on survival when added to the model (*p* = 0.20 for MM and *p* = 0.19 for MGUS, respectively).

In a sensitivity analysis, where mortality was compared using age-matched MM or MGUS controls, the results were almost identical (data not shown).

### Analyses by specific autoimmune disease

Of the included MM patients, 151 had a prior history of rheumatoid arthritis, 100 of pernicious anemia, 76 of chronic rheumatic heart disease, 52 of ulcerative colitis, 223 of polymyalgia rheumatica, 107 of psoriasis, and 58 of giant cell arteritis.

The increased risk of dying after ulcerative colitis was greater in MM patients (HR = 1.4, 95 % CI 1.0–1.9) than in controls (HR = 1.2, 95 % CI 0.9–1.7). For the other specific conditions analyzed, the excess mortality associated with a prior autoimmune disease was not different, or was lower, in MM patients compared to controls (Table 3).

**Table 3:** History of specific autoimmune conditions and survival in MM and MGUS (both genders)

	MM^a^			MGUS^b^		
	No. of MM patients with AI^c^ (%)	HR^d^	95 % CI^e^	No. of MGUS patients with AI (%)	HR	95 % CI
Rheumatoid arthritis	151 (1.8)			665 (3.5)		
MM/MGUS + AI vs. MM/MGUS + no AI	100/151	1.3	1.0–1.6	322/665	1.3	1.2–1.5
Controls + AI vs. controls + no AI	182/515	1.8	1.6–2.1	545/1334	1.8	1.7–2.0
Pernicious anemia	100 (1.2)			149 (0.8)		
MM/MGUS + AI vs. MM/MGUS + no AI	70/100	1.2	1.0–1.5	90/149	1.5	1.2–1.9
Controls + AI vs. controls + no AI	62/127	2.1	1.7–2.7	211/328	2.0	1.7–2.3
Chronic rheumatic heart disease	76 (0.9)			203 (0.1)		
MM/MGUS + AI vs. MM/MGUS + no AI	41/76	0.9	0.7–1.3	86/203	1.4	1.1–1.7
Controls + AI vs. controls + no AI	78/305	1.6	1.3–2.0	193/599	2.0	1.8–2.4
Ulcerative colitis	52 (0.6)			153 (0.8)		
MM/MGUS + AI vs. MM/MGUS + no AI	33/52	1.4	1.0–1.9	47/153	1.1	0.8–1.5
Controls + AI vs. controls + no AI	38/191	1.2	0.9–1.7	87/327	1.6	1.3–2.0
Polymyalgia rheumatica	223 (2.7)			817 (4.4)		
MM/MGUS + AI vs. MM/MGUS + no AI	146/223	1.0	0.8–1.2	343/817	1.0	0.9–1.2
Controls + AI vs. controls + no AI	227/603	1.5	1.4–1.8	600/1410	1.3	1.2–1.5
Giant cell arteritis	58 (0.7)			228 (1.2)		
MM/MGUS + AI vs. MM/MGUS + no AI	38/58	0.8	0.6–1.1	92/228	1.0	0.8–1.2
Controls + AI vs. controls + no AI	53/150	1.4	1.1–1.8	141/300	1.4	1.1–1.6
Psoriasis	107 (1.3)			336 (1.8)		
MM/MGUS + AI vs. MM/MGUS + no AI	51/107	0.9	0.7–1.2	103/336	1.3	1.0–1.6
Controls + AI vs. controls + no AI	98/507	1.3	1.1–1.6	203/824	1.4	1.2–1.6

^a^ *MM* multiple myeloma

^b^ *MGUS* monoclonal gammopathy of undetermined significance

^c^ *AI* autoimmune disease

^d^ *HR* hazard ratio

^e^ *CI* confidence interval

Of the included MGUS patients, 665 had a prior history of rheumatoid arthritis, 149 of pernicious anemia, 203 of chronic rheumatic heart disease, 153 of ulcerative colitis, 817 of polymyalgia rheumatica, 336 of psoriasis, and 228 of giant cell arteritis. For all conditions analyzed, the excess mortality added by a prior autoimmune disease was lower in MGUS patients than the increased risk of dying in controls with a prior autoimmune disease (Table 3).

### Analyses on MGUS patients by M-protein isotype and concentration

Information on M-protein isotype was available for 4210 individuals (22 %). The effect of a history of autoimmune disease on survival was not different between isotype IgA or IgG and isotype IgM.

Information on M-protein concentration was available for 3428 individuals (18 %). MGUS patients with a concentration of M-protein at diagnosis of 1.5 g/dL or more had a significantly higher risk of death (HR = 1.2, 95 % CI 1.0–1.4) compared to MGUS patients with a lower M-protein concentration. The interaction between autoimmune disease and concentration was not significant (*p* = 0.28).

## Discussion

In this large population-based study including over 8000 MM patients, almost 19,000 MGUS patients, and their 110,000 matched control subjects, we found that a history of autoimmune disease was associated with a reduced survival in MM and MGUS patients. Furthermore, although based on smaller numbers, ulcerative colitis had a greater impact on mortality in MM than other autoimmune conditions. These novel findings suggest a more aggressive disease course in autoimmunity-associated plasma cell disorders and that more attention should be paid to comorbidity when predicting prognosis and tailoring therapy.

The finding that autoimmune disease is a predictor of worse survival in MM patients is an important finding, and contradicts results from a previous smaller study on autoimmune disease and MM survival where no effect on survival in MM was observed [22]. However, the abovementioned study was a cohort study on individuals with autoimmune disease, and captured only 457 cases of multiple myeloma. Our findings are consistent with autoimmune disorders being a predictor of poor survival in the population [23, 24], and expand on previous investigations showing autoimmune disease to have negative impact on survival in Hodgkin lymphoma and in certain digestive tract cancers [17, 25]. The intensity of medical treatments for MM patients requires careful consideration of risks and benefits, particularly if there are other co-existing serious illnesses. With an aging population, the number of MM patients has grown and will continue to grow in the years to come [32]. In MM patients, comorbid diseases may increase the risk of treatment-related complications. Also, MM itself or its treatment may aggravate existing comorbid diseases, leading to lower performance status, decreased quality of life, and a shorter overall survival [33]. Despite the importance of considering comorbid diseases in the treatment and prognosis of MM, the literature on comorbidities in MM is limited and based on small series [34–38]. In these studies, survival is negatively influenced by increasing number of comorbid conditions. Approximately 80 % of MM patients have one or more comorbid conditions, and almost half have two or more [34–36]. There are few clinical trials where the elderly frail patients are included [39]; therefore, population-based studies are a valuable tool to estimate survival in a diverse MM population [32, 40–43].

In patients with MGUS, a history of autoimmune disease and its impact on survival have, to our knowledge, not been investigated previously. We found that MGUS patients with previous autoimmune disease had a significantly 1.4-fold increased risk of death. In patients with MGUS, the effect of a history of autoimmune disease on survival was not affected by isotype or by M-protein concentration at MGUS diagnosis. Previous investigators of cancer survival after autoimmune diseases have speculated that the underlying explanation may be poorer performance status or non-tolerance of therapy. However, this does not explain the decreased survival after autoimmune diseases in MGUS patients, who are asymptomatic by definition and do not receive therapy. Our findings of an increased risk of death in MGUS patients with a history of autoimmune disease thus suggest an unknown underlying factor which may impact the risk of death in MGUS patients with a prior autoimmune disease, and could also potentially shed light on the pathogenesis of MGUS. In addition, whether a prior autoimmune disease is also a risk factor for progression in MGUS needs to be clarified.

Considering autoimmune diseases are known to be more common in females than in males, we explored survival outcomes by sex to see if autoimmunity might be associated with different survival patterns for male and female patients with MM or MGUS; however, they were not.

Interestingly, a history of ulcerative colitis had a greater impact on survival in MM patients than a history of ulcerative colitis had in the general population, although the confidence intervals overlapped so the difference was not statistically significant. It is possible that therapy-related factors might have played a role. Another possibility is that individuals developing MM after ulcerative colitis are more likely to harbor additional poor prognostic factors.

Our study has several strengths, such as its large size and high-quality data from Sweden. The data is derived from a stable population with access to standardized medical health care during the entire study period, ensuring a generalizability of findings. The large study size has generated a high power, as shown in the narrow confidence intervals. Recall bias was ruled out due to the study design using nationwide registers. Because of the large study size, patient-related information was only gathered through registers, and we were not able to validate individual medical records, and we did not have information on established risk factors such as genetic aberrations detectable by fluorescence in situ hybridization (FISH), both of which are limitations of our study. Also, this is a hypothesis-generating study, including many autoimmune diseases, and the findings on specific autoimmune diseases should be interpreted with caution. In our study, individuals with a history of autoimmune disease were older than those without. However, analyses were adjusted for age, and a sensitivity analysis where mortality was compared between cases and age-matched controls showed almost identical results. Thus, the age difference is unlikely to explain the difference in survival. The results for MGUS are cohort-based, and although large, it does not necessarily represent the true population since MGUS is an asymptomatic condition and the cohort was clinically based, not a screened population. The use of inpatient data could have led to under-ascertainment of less severe forms of autoimmune diseases. Importantly, the autoimmune disease did not have to be the reason for admission, so all patients with autoimmune disease admitted for any reasons are included in our analysis. The prevalence of autoimmune disease was higher in MM (16 %) and MGUS (21 %) patients than in controls (12–13 %), which is consistent with previous findings of a significantly increased risk of MGUS and to some extent MM after autoimmune disease [20–22]. The overall high prevalence of autoimmunity in our study is surprising; however, a wide range of autoimmune disorders were included (Appendix 1) and although we were not able to validate individual medical records, since history of autoimmune diseases was assessed in the same way among MM and MGUS patients and matched controls, any under- or over-diagnosis should be non-differential.

In conclusion, our findings that a personal history of autoimmune disease has a negative impact on survival in MM and MGUS patients could be due to underlying common genetic factors, or that patients with a personal history of autoimmunity develop more severe forms of MM or MGUS as a result of either the autoimmune disease or its treatment, or cumulative comorbidity in the individual. Our findings suggest that more attention should be paid to comorbidity, such as autoimmune disease, as a prognostic factor in MM, and perhaps also in MGUS. Treatment options for MM are investigated today in randomized clinical trials which are subject to selection bias with strict inclusion and exclusion criteria, often with omission of elderly patients and patients with comorbidites. However, a majority of MM patients suffer from multiple other diseases, which may both affect survival and make patients unsuitable for certain treatments. Our findings raise interesting questions on the pathogenesis of MGUS, and highlight the importance of accounting for comorbidities such as autoimmune disease both for predicting prognosis and for tailoring therapy in patients with plasma cell dyscrasias.

## Appendix

**Table 4:** Autoimmune conditions included in the study

Rheumatoid arthritis	Lupoid hepatitis
Systemic sclerosis	Celiac disease
Sjögren’s syndrome	Granulomatosis with polyangiitis (Wegener’s)
Systemic lupus erythematosus	Dressler syndrome
Polymyositis or dermatomyositis	Chronic rheumatic heart disease
Hashimoto thyroiditis	Multiple sclerosis
Grave’s disease	Amyotrophic lateral sclerosis
Addison disease	Rheumatic fever
Pernicious anemia	Sarcoidosis
Autoimmune hemolytic anemia	Reiter disease
Immune thrombocytopenia	Crohn disease
Primary biliary cirrhosis	Ulcerative colitis
Discoid lupus erythematosus	Ankylosing spondylitis
Localized scleroderma	Polymyalgia rheumatica
Myasthenia gravis	Psoriasis
Autoimmune hepatitis	Behcet disease
Polyarteritis nodosa	Giant cell arteritis
Guillain-Barré syndrome	Vitiligo
Diabetes type 1	Aplastic anemia
